# The Surgical Burden of Animal Bites Referred to Orthopaedics: A 10-Year Cohort Study of 168 Patients From a UK Major Trauma Centre

**DOI:** 10.7759/cureus.97490

**Published:** 2025-11-22

**Authors:** Nicholas Tse Hao Ng, Khemerin Eng, Joseph Northway, Alan R Norrish

**Affiliations:** 1 Department of Orthopaedics, Queen’s Medical Centre, Nottingham University Hospitals NHS Trust, Nottingham, GBR; 2 Department of Orthopaedics, Queen's Medical Centre, Nottingham University Hospitals NHS Trust, Nottingham, GBR; 3 Department of Trauma and Sports Medicine, School of Medicine, Queen's Medical Centre, University of Nottingham, Nottingham, GBR

**Keywords:** animal bite management, cat bite, complication, dog bites, epidemiology and public health, major trauma centre, microbiological profile, musculoskeletal injury, surgical burden, trauma and orthopaedics

## Abstract

Introduction

Animal bites impose a substantial, yet under-recognised, burden on orthopaedic services. We characterised the epidemiology, injury patterns, management, and short-term outcomes of bite injuries requiring orthopaedic input at a UK Major Trauma Centre over 10 years.

Methods

We performed a retrospective cohort study of consecutive patients referred to orthopaedics with animal-bite injuries between December 2015 and March 2025. Eligible cases were identified from a prospectively maintained trauma database and verified against electronic records. Inclusion encompassed mammalian, reptile, avian, fish, amphibian, and spider bites; human, “fight,” and insect stings/tick exposures were excluded. Demographics, injury site and structures involved, management (operative vs non-operative), microbiology, complications, readmissions, and length of stay (LOS) were extracted using a standardised proforma. Early presentation was defined as <24 hours post-injury. Descriptive statistics were used.

Results

We included 168 patients. A total of 158 (94.0%) were adults; 89 (53.0%) were females; and the mean age was 49 years. Early presentation occurred in 88 (52.4%); LOS 2.6 vs 2.9 days for delayed. Domestic animals accounted for 166 (98.8%) injuries, dogs (110, 65.5%) and cats (56, 33.3%). Upper limb involvement predominated, 160 (95.2%); hand injuries were most common, 128 (76.2%). Structural damage included 11 (6.5%) open fractures, 15 (8.9%) tendon, 14 (8.3%) muscle, seven (4.2%) nerve, and two (1.2%) vascular injuries. Dog bites accounted for all open fractures and all neurovascular injuries, and most tendon (11, 73.3%) and muscle (11, 78.6%) trauma; multi-limb injuries were dog-related in 16 (88.9%). Overall, 109 (64.9%) underwent surgery and 12 (7.1%) required reoperation. Microbiology was obtained in 82 (48.8%); 41 (50.0%) were culture-negative. *Pasteurella *spp. predominated, *Pasteurella canis* in dog bites and *Pasteurella multocida* in cat bites, frequently associated with debridement. Readmissions were more common after delayed presentation (5 vs 2), including one digit amputation.

Conclusions

Over a decade, animal bites referred to orthopaedics were chiefly domestic, concentrated in the hand, and often required surgery, particularly dog-bite injuries with complex structural damage. Early presentation shortened LOS but did not avert operative need; delayed presentation carried higher infectious morbidity. Findings support prompt assessment, species-informed empirical antibiotics, and streamlined orthoplastic pathways, alongside public-health measures promoting early care-seeking and responsible ownership.

## Introduction

Animal bites impose a substantial, yet under-recognised, burden on orthopaedic services, encompassing severe soft-tissue and bony injury, infection, and long-term functional impairment. Recent public attention, stimulated by the Department for Environment, Food and Rural Affairs (DEFRA) ban on XL Bullies, a powerful breed implicated in multiple fatal attacks in the UK, has renewed focus on the scale and consequences of such injuries [1]. However, most epidemiological research centres on emergency department attendance, leaving a critical gap in understanding the downstream orthopaedic workload, particularly cases necessitating specialist operative intervention.

Globally, the World Health Organisation identifies snakes and dogs as leading causes of bite injury, estimating approximately five million snake bites and tens of millions of dog bites annually [2]. Kennedy et al. suggest that roughly half of the population will experience an animal bite during their lifetime, with domestic animals responsible for the majority [3]. True incidence remains uncertain: international estimates are hampered by reliance on hospital-based data, and many individuals neither report nor seek care for bite injuries. In the UK, under-ascertainment is substantial; a cross-sectional survey by Westgarth et al. found that up to 60% of bites, particularly those from household pets, go unreported [4]. Despite this underreporting, recent NHS data indicate a 47% rise in dog-bite admissions since 2018, with around 8,000 admissions annually and approximately 3,000 requiring operative management [5]. These figures imply a sizeable orthopaedic impact that is not well captured in current literature.

This study addresses that gap by characterising the orthopaedic burden of animal bites presenting to a UK Major Trauma Centre over a 10-year period. Our primary aim is to describe the epidemiology, management, and outcomes of bite-related injuries requiring orthopaedic input. Specific objectives are to: (i) quantify the operative caseload; (ii) delineate demographic patterns, injury mechanisms (including species), anatomical distribution, and fracture involvement; (iii) describe timelines to presentation, microbiology, antimicrobial therapy, and debridement strategies; (iv) evaluate short-term outcomes including infection, readmission, re-operation, and length of stay; and (v) estimate service implications for theatre utilisation and follow-up. By focusing on orthopaedic endpoints rather than initial attendance alone, this work seeks to inform clinical pathways, resource planning, and prevention strategies in line with national priorities for patient safety and theatre efficiency.

## Materials and methods

We conducted a retrospective cohort study of all animal-bite injuries referred to the orthopaedic service at Queen’s Medical Centre, Nottingham, a UK Major Trauma Centre, between December 2015 and March 2025. This study was approved by the Nottingham University Hospitals NHS Trust Clinical Effectiveness Team, Nottingham, UK (approval number: 24-989C; date: March 18, 2025). Eligible cases were identified from a prospectively maintained trauma database and cross-checked against electronic health records to ensure completeness. The study population comprised all patients of any age referred to orthopaedics with an injury attributable to an animal bite. Bites from mammals, reptiles, birds, fish, and amphibians were included, as were spider bites. Other arachnid and insect exposures (e.g. stings and tick bites) were excluded to avoid cohorts whose primary issues are toxicological or immunological, such as anaphylaxis or zoonotic transmission, rather than mechanical soft-tissue or bony trauma likely to require orthopaedic assessment. Human bites and “fight bites” were excluded. Cases managed solely in the emergency department or by other specialties without orthopaedic input, and those with materially incomplete records, were also excluded.

Data were extracted using a standardised proforma. A structured search strategy was applied to diagnostic fields and clinical notes, using keyword stems “bite*” and “animal*” to identify candidate cases, followed by manual screening against inclusion and exclusion criteria. Demographic variables included age, sex, and comorbidity profile, with a priori emphasis on diabetes. Adults were defined as individuals aged ≥18 years. Injury characteristics captured the biting species, time from injury to presentation, anatomical site, depth and structures involved (skin, subcutaneous tissue, tendon, nerve, vessel, bone), and available microbiology (culture results and organism profile).

Management was classified as operative or non-operative. Operative care was defined as any procedure undertaken in theatre (e.g. washout, debridement, exploration, repair, or fixation). Non-operative (conservative) management comprised limb elevation, analgesia, and antimicrobial therapy delivered orally or intravenously according to clinical judgement and local policy. Allied specialty involvement (e.g. plastics, infectious diseases, microbiology) and the total number and type of procedures per episode were recorded. Complications were prospectively coded from the medical record.

Outcomes included length of stay, 30-day readmission, surgical-site or wound infection, and reoperation. Infection was defined as the presence of purulent discharge with positive culture, or clinical features prompting antibiotic escalation and/or further debridement, as documented by the treating team. Data handling followed routine clinical governance procedures; no patient-identifiable information was exported for analysis. Continuous variables are reported using standard summary statistics, and categorical variables as counts and percentages.

## Results

Cohort characteristics

Over the 10-year period, 168 patients met the inclusion criteria (Table 1). Most were adults (n=158, 94.0%), with a slight female predominance (n=89, 53.0%). The mean age was 49 years (median 52; IQR: 35.8-60.25). Twelve patients (7.1%) had diabetes. The average interval from injury to hospital presentation was two days (range 0-14), and 88 (52.4%) patients presented early, defined as within 24 hours of injury. Mean length of stay (LOS) for the whole cohort was 2.7 days (SD ±2.75; range 0-21). Early presenters had a marginally shorter LOS than delayed presenters (2.6 vs 2.9 days; SD ±2.86 and ±2.65, respectively). Early presenters more frequently underwent surgery (69/88, 78.4%) than delayed presenters (40/80, 50.0%). Six patients in each subgroup required a planned second-look procedure or repeat debridement. Conservative treatment failed in four early presenters and one delayed presenter. Readmission occurred in two early presenters (failed conservative management or wound necrosis) and in five delayed presenters (three abscesses, two osteomyelitis), one of whom subsequently required amputation of a digit (Table 1).

**Table 1:** Cohort characteristics and outcomes. Early presentation defined as arrival <24 hours post-injury. Length of stay (LOS) reported as mean (SD); overall LOS also includes range. “Operative management” refers to any procedure performed in theatre. “Second-look/repeat debridement” includes planned re-exploration or return to theatre for further debridement. Percentages in subgroup columns use the subgroup denominator (n=88 early; n=80 delayed). Dashes indicate data not reported for that subgroup.

Cohort characteristics and outcomes	Overall (N=168)	Early presenters (<24 h, n=88)	Delayed presenters (≥24 h, n=80)
Adults, n (%)	158 (94.0)	—	—
Female, n (%)	89 (53.0)	—	—
Age, years; mean; median (IQR)	49; 52 (35.8-60.25)	—	—
Diabetes, n (%)	12 (7.1)	—	—
Injury time → presentation; days, mean (range)	2 (0-14)	—	—
Presented within 24 h, n (%)	88 (52.4)	88 (100)	—
Length of stay, days; mean (SD; range)	2.7 (2.75; 0-21)	2.6 (2.86)	2.9 (2.65)
Operative management, n (%)	—	69 (78.4)	40 (50.0)
Second-look/repeat debridement, n	—	6	6
Failed conservative management, n	—	4	1
Readmissions, n (reason)	7 (see subgroups)	2 (failed conservative; wound necrosis)	5 (3 abscess; 2 osteomyelitis)
Amputation related to readmission, n	1 (digit)	0	1 (digit)

Animal type

**Figure 1:**
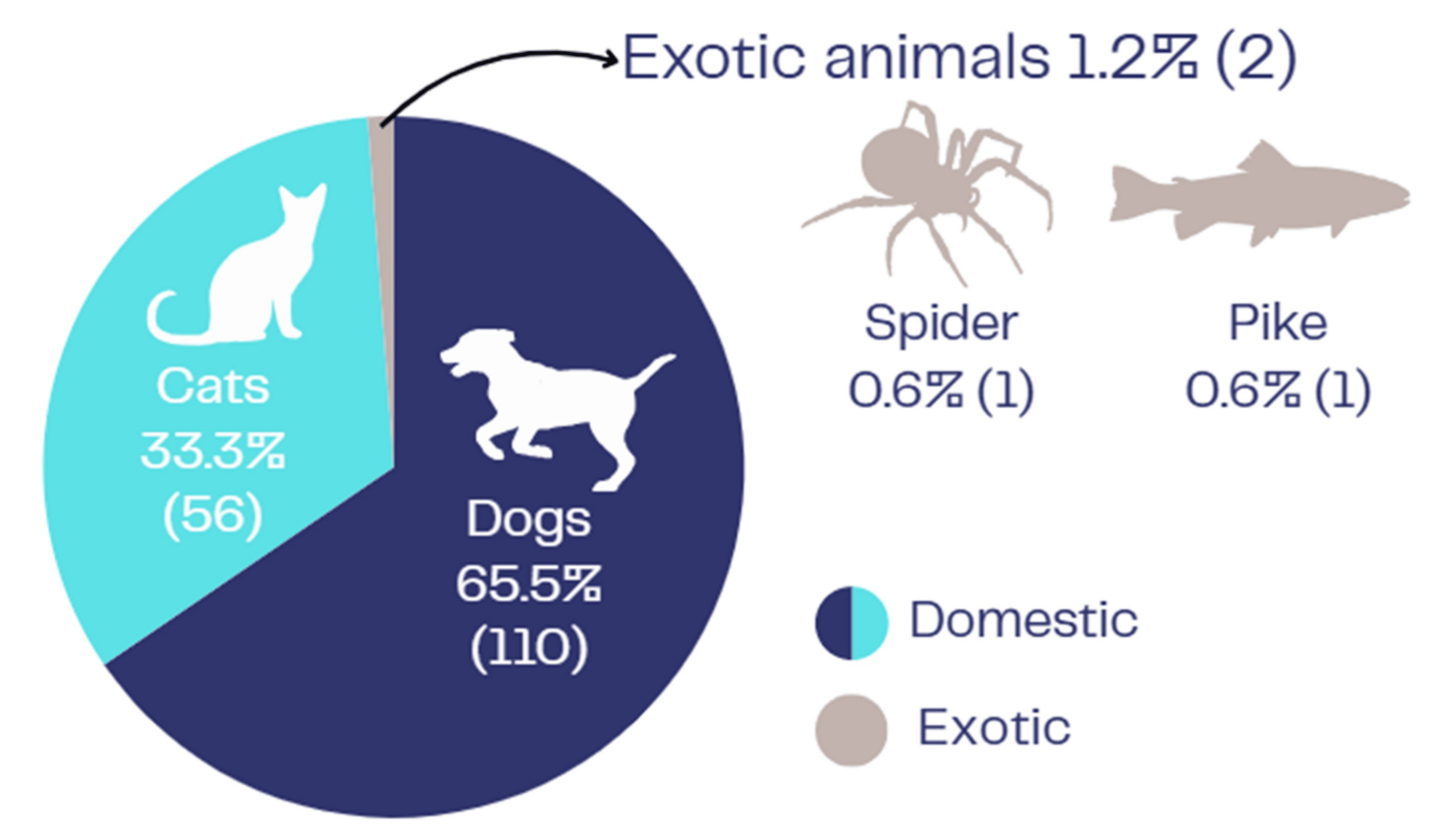
Animal types responsible for the bites. All blue shades were domestic animals. Designed by author Nicholas Ng.

Injuries were overwhelmingly caused by domestic animals (n=166, 98.8%), with dogs responsible for 65.5% (n=110) and cats for 33.3% (n=56) of cases; two injuries (1.2%) followed bites from exotic species, one spider and one pike (Figure 1).

Injury characteristics

Upper limb involvement predominated (160/168, 95.2%), most commonly the hand (n=128, 76.2%) and forearm (n=25, 14.9%). Lower limb injuries were less frequent (n=13, 7.7%), with the leg the most commonly affected site (n=6, 3.6%). Eighteen (10.7%) patients sustained multi-limb injuries, and 16 (88.9%) following dog bites. Two (1.2%) patients had associated chest or abdominal trauma in addition to their orthopaedic injuries (Table 2). Among hand injuries (defined as distal to the carpus), 80/128 (62.5%) followed dog bites, 47/128 (36.7%) cat bites, and one was due to a spider bite.

**Table 2:** Injury distribution and structural damage. Percentages are calculated using the full cohort denominator (N=168) unless otherwise specified in the breakdown column. Subtotals within categories may not sum to category totals due to multiple sites per patient.

Injury distribution	n	% of cohort (N=168)
Anatomical region(s)		
Upper limb (any)	160	95.2
Hand	128	76.2
Forearm	25	14.9
Wrist	13	7.7
Elbow	5	3.0
Arm	2	1.2
Lower limb (any)	13	7.7
Leg	6	3.6
Ankle	3	1.8
Thigh	2	1.2
Knee	1	0.6
Foot	1	0.6
Multi-limb injuries	18	10.7
Associated chest/abdominal trauma	2	1.2
Structural damage		
Open fractures	11	6.5
Tendon injuries	15	8.9
Muscle injuries	14	8.3
Peripheral nerve injuries	7	4.2
Vascular injuries	2	1.2

Structural injury was common (Table 2). Open fractures occurred in 11 (6.5%) patients: six distal radius or ulna fractures, one metacarpal fracture, and four phalangeal fractures. Tendon injuries were recorded in 15 (8.9%) patients, eight of which involved the flexor sheath of the hand. Muscle trauma affected 14 (8.3%) patients, typically the forearm flexor and extensor bellies or the thenar eminence. Peripheral nerve injury occurred in seven (4.2%) patients, most often involving the ulnar nerve or radial digital nerves. Two (1.2%) patients had vascular injuries, one to the ulnar artery and one to the ulnar digital artery.

Dog bites

Among the 110 dog-bite patients, eight (7.3%) were children (Table 3). Dog bites accounted for all open fractures, all peripheral nerve and vascular injuries, and the majority of tendon (11/15, 73.3%) and muscle injuries (11/14, 78.6%). Multi-limb involvement was also largely dog-related (16/18, 88.9%). Severe injuries predominantly affected adults: all tendon, muscle, and neurovascular injuries, and 10 of 11 open fractures, occurred in adult patients.

**Table 3:** Dog-bite cohort: characteristics, injury severity, and management. Percentages shown in the second numeric column use the dog-bite denominator (N=110). Subtotals may not sum due to overlapping categories (e.g., patients with both tendon and nerve injury). Total surgically treated patients include patients managed initially by surgery and patients requiring surgery following failed conservative management.

Dog-bite cohort: characteristics, injury severity, and management	n	% within dog-bite cohort (N=110)
Demographics		
Adult	102	92.7
Children	8	7.3
Injury severity profile		
Open fractures attributable to dog bites	11	10.0
Peripheral nerve injuries attributable to dog bites	7	6.5
Vascular injuries attributable to dog bites	2	1.8
Tendon injuries attributable to dog bites	11	10.0
Muscle injuries attributable to dog bites	11	10.0
Multi-limb injuries after dog bites	16	14.5
Initial management		
Conservative (antibiotics, elevation)	34	30.9
Surgical management		
Total surgically treated patients	78	70.9
Combined orthoplastic procedures	11	10.0
Additional general surgical input	1	0.9
Multiple operations (≥2 theatre episodes)	8	7.3
Postoperative infection or wound necrosis	4	3.6
Other postoperative complications	3	2.7

Initial conservative management (antibiotics and elevation) was instituted in 34/110 (30.9%) dog-bite cases. Complications occurred in three (8.8%) conservatively managed patients: one developed anaphylaxis to co-amoxiclav (first-line local therapy), and two experienced clinical deterioration requiring surgical debridement; one of these subsequently developed postoperative hand compartment syndrome necessitating emergency fasciotomy and a return to theatre. The remaining 76 patients underwent urgent surgery. Combined orthoplastic procedures were required in 11/78 (14.1%) surgically treated patients, and one polytraumatised patient needed additional general surgical input for abdominal injuries alongside orthoplastic care. Eight (10.3%) patients required multiple operations; one underwent four returns to theatre after the index debridement. Postoperative infection or wound necrosis occurred in four (5.1%) patients; three required further debridement and one was managed successfully with antibiotics. Other postoperative complications, functional weakness, chronic stiffness, and acute kidney injury, were each observed in separate patients (3/78, 3.8%) (Table 3).

Cat bites

Cat bites predominantly involved the upper limb (55/56, 98.2%), especially the hand (47/56, 83.9%) (Table 4). Four tendon injuries were documented (two flexor sheath, two forearm extensors), and three patients had muscle injury, two affecting the thenar eminence. Conservative management was used in 30/56 (53.6%); five (16.7%) failed conservative therapy and required operative intervention. Among the 26 patients who underwent urgent debridement, two (7.7%) developed postoperative infection requiring readmission and further procedures. Overall, 31 patients had operations; three (9.7%) required orthoplastic input, and three (9.7%) underwent more than one operative episode. Two postoperative infections necessitated further debridement; one culminated in distal phalanx amputation of the index finger due to osteomyelitis (Table 4).

**Table 4:** Cat-bite cohort: distribution, management, and outcomes. Percentages are calculated with the cat-bite denominator (N=56). Subtotals may not sum because categories can overlap (e.g., multisite injury in the same patient).

Cat-bite cohort: distribution, management, and outcomes	n	% within cat-bite cohort (N=56)
Anatomical distribution		
Upper limb (any)	55	98.2
Hand	47	83.9
Wrist	7	12.5
Forearm	3	5.4
Ankle	1	1.8
Tissue/structural injury		
Tendon injury	4	7.1
Muscle injury	3	5.4
Initial management		
Conservative treatment (antibiotics ± elevation)	30	53.6
Failure of conservative therapy → surgery	5	8.9
Urgent debridement (index operation)	26	46.4
Surgical outcomes		
Any operation during episode of care	31	55.4
Orthoplastic input required	3	5.4
Multiple operations (≥2 theatre episodes)	3	5.4
Complications		
Postoperative infection requiring readmission	2	3.6
Further debridement for postoperative infection	2	3.6

Microbiology

Intraoperative or wound microbiology was obtained in 82/168 patients (48.8%) (Table 5). All patients received empirical antibiotics on presentation, prior to sampling. Half of all samples yielded no growth after prolonged incubation (41/82, 50.0%). Among dog-bite samples (n=49), 24 (49.0%) were culture-negative. The most frequently isolated organism was *Pasteurella *spp. (n=8, 16.3%), followed by *Staphylococcus aureus* (n=5, 10.2%), Group G Streptococcus (n=3, 6.1%), and *Neisseria *spp. (n=3, 6.1%). Other organisms included coliforms, *Enterobacter ludwigii*, *Pseudomonas aeruginosa*, *Bacteroides pyogenes*, and *Bergeyella zoohelcum*. *Pasteurella canis* was the most common *Pasteurella* species recovered from dog-bite wounds, and all Pasteurella-positive cases underwent surgical debridement. Mixed growth or polymicrobial cultures were present in six samples (12.2%).

**Table 5:** Microbiological profile. Percentages are calculated using the denominator of each subgroup as indicated in the breakdown column. Subtotals within categories may not sum to category totals due to some samples being polymicrobial.

Microbiological profile: distribution and culture results	n	% within subgroups
Sampled cohort		
Total samples	82	100.0
No growth	41	50.0
Positive cultures	38	46.3
Rejected samples	3	3.7
Dog bites		
Subgroup total	49	100.0
No growth	24	49.0
Positive culture	23	46.9
*Pasteurella *spp.	8	16.3
Staphylococcus aureus	5	10.2
Group G Streptococcus	3	6.1
Neisseria spp.	3	6.1
Mixed growth/ polymicrobial	6	12.2
Rejected samples	2	4.1
Cat bites		
Subgroup total	31	100.0
No growth	16	51.6
Positive culture	14	45.2
Pasteurella multocida	10	32.3
Rejected samples	1	3.2
Exotic bites		
Subgroup total	2	100.0
No growth	1	50.0
Positive culture	1	50.0

**Figure 2:**
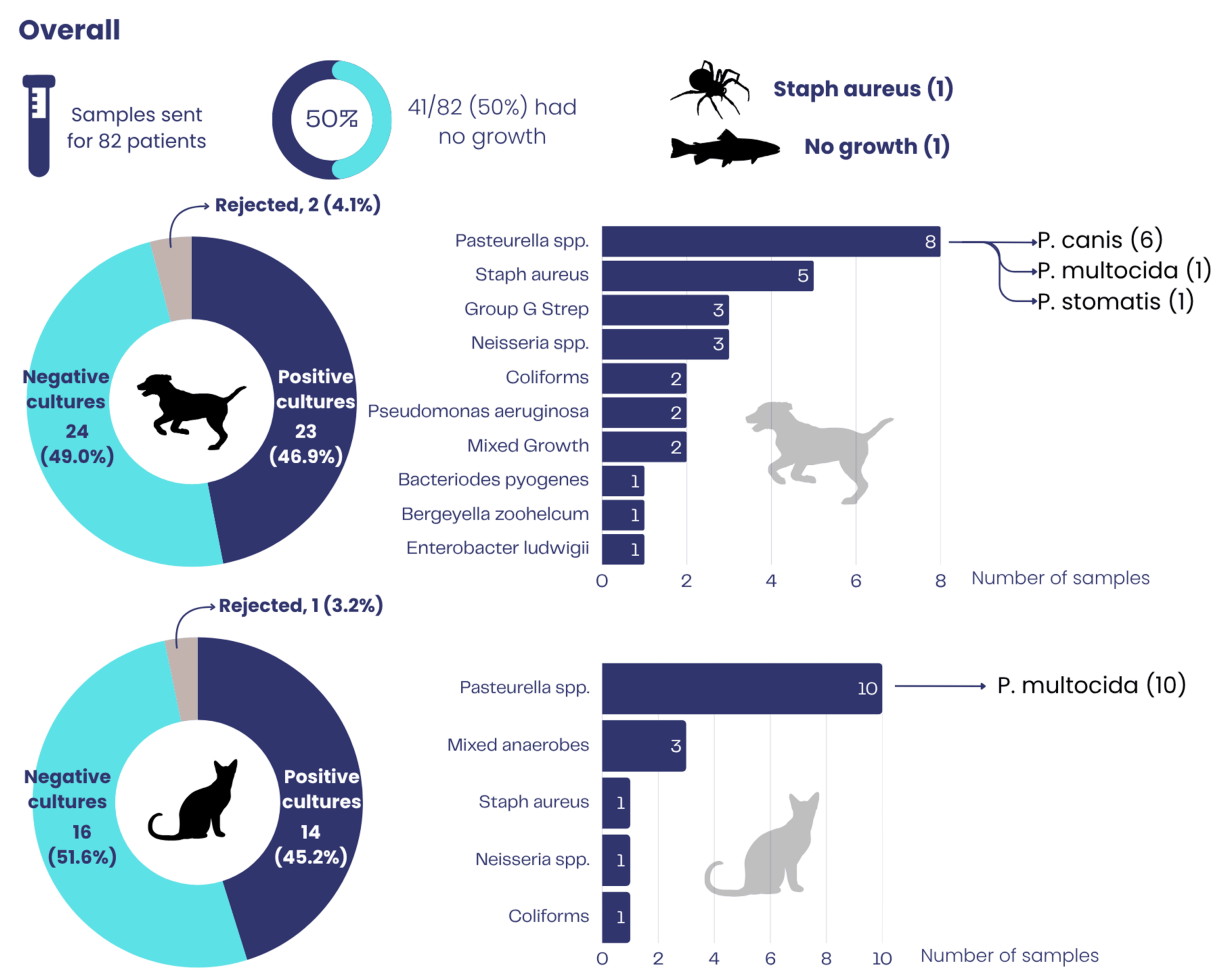
Microbiology of animal-bite wounds. Subtotals of organism types may not sum to sample count due to some samples being polymicrobial (e.g. *Staphylococcus aureus* and *Pasteurella *spp. in the same patient). Designed by author Nicholas Ng.

Among cat-bite samples (n=31), 16 (51.6%) were culture-negative and one sample was rejected. Of the 14 positive cultures, *Pasteurella multocida* predominated (10/14, 71.4%); eight of these 10 cases required surgical debridement. Additional isolates included *Staphylococcus aureus*, coliforms, *Neisseria *spp., and mixed anaerobes. The spider-bite case yielded *Staphylococcus aureus* from an intraoperative hand abscess specimen, whereas the pike-bite wound cultured no organisms (Figure 2).

Infographic

**Figure 3:**
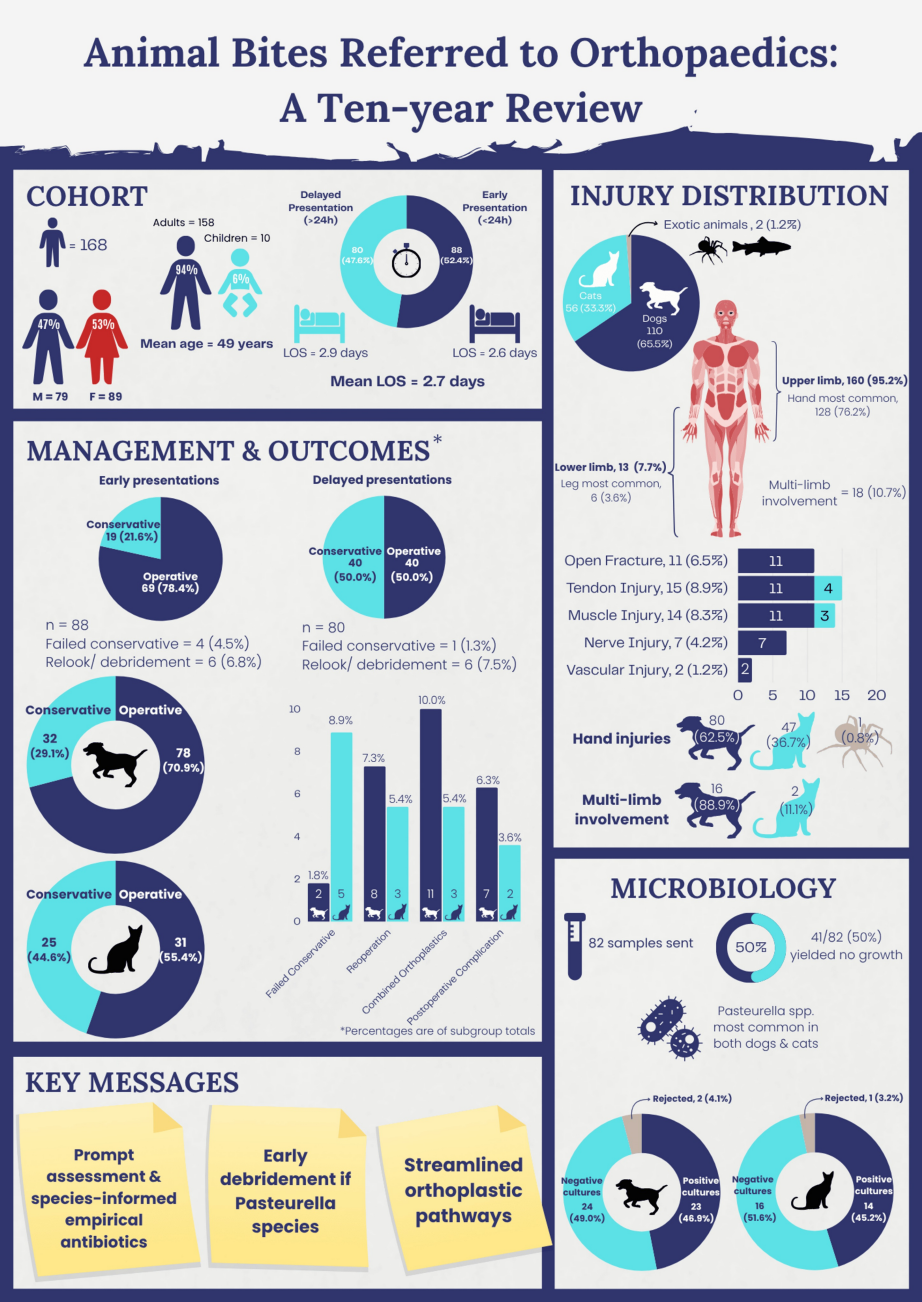
Infographic of principal findings. A visual summary of the epidemiology, injury characteristics, microbiological profile, management, and clinical outcomes of 168 animal bite injuries managed by a UK Major Trauma Centre orthopaedic service (2015-2025). Designed by author Nicholas Ng. LOS: length of stay.

Figure 3 provides a graphical summary of the principal findings from this study.

## Discussion

Across a decade at a UK Major Trauma Centre, animal bites referred to orthopaedics were overwhelmingly domestic in origin and principally involved the hand. Dog bites were associated with the greatest injury severity, accounting for all open fractures and all neurovascular injuries, and the majority of tendon and muscle trauma. Early presentation (<24 hours) was common and associated with a slightly shorter length of stay; however, operative management remained frequent, particularly among early presenters. Microbiologically, culture-negative results were common, yet *Pasteurella* species, especially *P. canis* in dog bites and *P. multocida* in cat bites, predominated among positive cultures and were closely linked with the need for surgical debridement (Table 5). These findings delineate a sizeable, procedurally intensive orthopaedic burden with distinct patterns by animal type.

Our results are consistent with national trends demonstrating a growing healthcare impact from animal bites, particularly those involving domestic dogs. Westgarth et al. reported rising hospital attendances attributable to dog bites in the UK, noting an annual incidence far exceeding earlier estimates [4]. Although only a small proportion of all bite incidents result in admission at the population level, our referred cohort shows a markedly higher surgical burden: nearly two-thirds required operative management and 7% underwent reoperation during the index admission. This aligns with, but also exceeds, figures reported in other series of hospital-managed bites, underscoring that orthopaedic referrals represent a severity-enriched subset.

Comparative international data highlight how regional exposures shape injury profiles. Tuncali et al. described a large series in which canine and feline bites likewise predominated, but with greater representation of other animals and a male preponderance [6]. In our cohort, females constituted a slight majority, and upper limb involvement, especially the hand, was dominant. These differences likely reflect variations in occupational exposure, domestic animal ownership patterns, and cultural norms of animal handling.

Disaggregation by species provides clinically important detail. In our series, dog bites caused all open fractures and all peripheral nerve and vascular injuries and were disproportionately linked to tendon and muscle belly damage. Similar observations have been made elsewhere: Van Eeckhout and Wylock reported that dog bites in adults were more likely to result in complex open injuries requiring vascular and plastic surgery input [7], while Stoclet et al. documented high rates of fracture (25%) and nerve injury (12%) among dog-bite victims across an 11-year single-centre experience [8]. By contrast, cat bites were overwhelmingly localised to the hand and frequently progressed despite initial conservative care. Wangler et al. found that a substantial fraction of cat-bite wounds cultured pathogens and required surgery [9], a pattern mirrored here, including one case culminating in digital amputation for osteomyelitis. Talan et al. similarly identified *Pasteurella *species as the leading pathogens in both dog and cat bites across multiple centres [10], and Babovic et al. showed that a notable minority of cat-bite patients deteriorate on conservative therapy and need operative intervention, occasionally more than once [11]. Collectively, these data reinforce a low threshold for surgical referral when deep structures of the hand may be involved.

Half of all samples in our cohort yielded no growth despite prolonged incubation. This likely reflects early empirical antibiotics before sampling, as suggested by Wangler et al. [9]. When organisms were isolated, *Pasteurella *species predominated, *P. canis* in dog bites and *P. multocida* in cat bites, consistent with Talan et al. [10]. All Pasteurella-positive wounds in our series underwent debridement, underscoring the clinical association between these pathogens and operative management. These findings support early antimicrobial therapy targeting Pasteurella alongside *Staphylococcus aureus* and streptococci, with prompt surgical assessment when there is suspicion of deep space infection, tendon sheath involvement, or necrotic tissue. Routine culture remains valuable, particularly in cases of clinical deterioration, failure of conservative measures, or atypical exposures.

Time to presentation was a key determinant of the clinical course. Early presenters were more likely to undergo surgery during the index admission, but delayed presenters exhibited higher rates of readmission, abscess formation, osteomyelitis, and amputation. This pattern echoes Seegmueller et al., who reported more complications and repeat operations among late presenters [12]. Benson et al. further demonstrated that a small subset of complex bite cases account for a disproportionate share of treatment costs, positing that earlier antibiotic therapy and wound care may reduce downstream procedural demand [13]. Public health messaging that encourages prompt hospital assessment, particularly for hand bites or in higher-risk patients (e.g. diabetes, neuropathy, immunosuppression), is therefore warranted.

Although the mean length of stay was modest, this likely underestimates true resource utilisation. Many patients require staged procedures, specialist orthoplastic input, rehabilitation, and close follow-up to monitor for late complications such as tendon adhesions, joint stiffness, or chronic infection. Multisite injuries, common in dog attacks, compound complexity; one patient in our cohort required multispecialty care for concomitant abdominal trauma. Centres without integrated orthoplastic services may face coordination challenges; our data support streamlined pathways that enable early combined assessment by orthopaedics, plastics, and microbiology when indicated.

The 2023 DEFRA ban on XL Bullies reflects national concern about severe dog attacks [1]. While our audit does not include breed-level data, the concentration of severe injuries within the dog-bite subset indicates that breed-specific surveillance could be informative for both prevention and legislation. Nonetheless, the capacity of any large dog to inflict devastating hand and limb injuries should not be underestimated. Prevention strategies must therefore extend beyond breed restrictions to encompass responsible ownership, training, supervision around children, and timely access to care.

Strengths of this study include its decade-long time frame, use of a prospectively maintained trauma database, and focus on orthopaedic endpoints most relevant to operative planning and resource allocation. The granularity of anatomical and structural injury detail by species, coupled with microbiological correlation, provides practical guidance for triage and initial management. Limitations must also be considered. The retrospective design relies on accurate documentation and coding. In our centre, emergency hand trauma is split between orthopaedics and plastic surgery according to weekday rota, so our dataset captures management of hand trauma under orthopaedic admissions; the true volume of hospital-managed hand bites is therefore likely higher. Patients managed entirely in the emergency department or solely by other specialties without orthopaedic involvement were excluded, potentially underestimating the denominator and skewing towards more severe cases. Breed data were unavailable, precluding evaluation of legislation effects such as the DEFRA ban [1]. Long-term functional outcomes, the durability of reconstruction, and comprehensive cost analyses were beyond scope. Finally, although we recorded time to presentation, qualitative reasons for delay, awareness, access barriers, or initial management in primary care, were not captured and merit prospective study.

From a practical standpoint, our findings support several important clinical implications. First, adopting a low threshold for early surgical assessment of hand bites, particularly dog bites, with attention to tendon sheath, deep space, and neurovascular injury is typically indicated. Second, ensuring empirical antibiotics are given that cover *Pasteurella*, *S. aureus*, and streptococci, with early escalation if clinical progress stalls or cultures indicate resistant organisms [6,10]. Third, coordinated orthoplastic pathways must be embedded for complex injuries and consideration given to developing standardised prompts for early imaging and compartment monitoring in high-risk dog bites. Lastly, strengthening discharge and follow-up protocols to detect late infection or functional decline, especially in patients who initially present late or who have comorbidities, is likely needed.

Future prospective studies should evaluate long-term functional outcomes, recurrence of infection, and patient-reported measures, alongside health-economic modelling to quantify the system-wide impact of bite injuries. A regional or national registry of surgically managed animal bites would enable breed-level risk assessment, benchmarking of operative strategies, and refinement of empirical antibiotic guidelines. Enhanced microbiological surveillance, particularly in failed conservative cases, could reduce reoperation through earlier targeted therapy. Finally, public health initiatives promoting responsible ownership, early presentation, and immediate wound care have the potential to reduce both incidence and severity [4,6,10-13].

## Conclusions

In a large, single-centre audit spanning 10 years, animal bites referred to orthopaedics were predominantly domestic, concentrated in the hand, and frequently required surgery. Dog bites carried the highest risk of complex structural injury and multisite trauma; cat bites, although often initially managed conservatively, commonly involved *P. multocida* and not infrequently progressed to operative debridement. Early presenters had higher rates of surgical intervention but fewer readmissions; late presenters experienced more infectious complications and readmissions. These findings underscore the need for prompt assessment, species-informed antimicrobial therapy, and coordinated orthoplastic care, while highlighting opportunities for prevention, surveillance, and targeted policy to lessen the orthopaedic burden of animal bite injuries.
